# Advanced MRI Sequences for Structural Lesion Assessment in Sacroiliitis

**DOI:** 10.3390/diagnostics16060887

**Published:** 2026-03-17

**Authors:** Törehan Özer, Emine Hafize Sönmez, Yonca Anik

**Affiliations:** 1 Department Radiology, Kocaeli University, İzmit 41380, Türkiye; yoncaanik@kocaeli.edu.tr; 2 Department of Pediatric Rheumatology, Kocaeli University, İzmit 41380, Türkiye; emine.sonmez@kocaeli.edu.tr

**Keywords:** back pain, sacroiliitis, MRI, 3D-MENSA, 3D-MERGE, ZTE, pediatric

## Abstract

**Background/Objectives:** Assessing structural damage in pediatric sacroiliitis is challenging, necessitating radiation-free alternatives to computed tomography (CT). This study evaluated the diagnostic performance of advanced MRI sequences—3D-MENSA (Multi-Echo in Steady-State Acquisition), 3D-MERGE (Multiple-Echo Recombined Gradient Echo), and Zero Echo Time (ZTE)—against conventional T1-weighted sequences for detecting structural lesions. Low-dose computed tomography (LDCT) served as the reference standard. A secondary objective was to qualitatively assess the visibility of active inflammatory lesions and fat metaplasia. **Methods:** In this cross-sectional study, 23 pediatric patients with enthesitis-related arthritis (ERA) were included. To adhere strictly to radiation safety principles, the study used pre-existing ldCT datasets from a clinical cohort as the reference standard. No new CT scans were performed for this study. Structural lesions (erosions, sclerosis, and joint-space changes) were independently scored by two blinded radiologists. Interobserver agreement was assessed using intraclass correlation coefficients (ICC). **Results:** Advanced sequences (ZTE, 3D-MENSA, 3D-MERGE) demonstrated high agreement with ldCT for erosion detection (ICC range: 0.924–0.998) and significantly outperformed conventional T1-weighted MRI (ICC: 0.707). 3D-MENSA provided distinct contrast, effectively differentiating the ligamentous component of the sacroiliac joint from both the synovial component and the adjacent bone cortex. Qualitatively, 3D-MENSA also identified bone marrow edema and fat metaplasia, which cannot be visualized by ZTE or ldCT. **Conclusions:** 3D-MENSA and 3D-MERGE enable comprehensive evaluation of structural sacroiliitis lesions in pediatric patients with diagnostic accuracy comparable to ldCT. Specifically, 3D-MENSA demonstrates the potential to detect both active and chronic lesions in a single, rapid, radiation-free acquisition. These findings suggest that it should be considered for routine pediatric imaging protocols.

## 1. Introduction

The sacroiliac joint (SIJ) is a complex, deep-seated articulation between the sacrum and the ilium, contributing significantly to load transmission and pelvic stability. Its involvement is a hallmark of spondyloarthropathies, including enthesitis-related arthritis (ERA), a subtype of juvenile idiopathic arthritis (JIA). However, assessing SIJ inflammation in pediatric populations is particularly challenging due to both clinical and imaging-related limitations. The pain associated with sacroiliitis is often poorly localized or referred to the lower back, buttocks, or thighs, making clinical suspicion difficult. Commonly used SIJ provocation tests, such as the FABER test and Gaenslen’s test, lack sensitivity and specificity in children and have not been validated for routine pediatric use [1]. Radiographically, conventional pelvic X-rays have limited value in early disease, because structural damage may take years to develop and become visible. Magnetic resonance imaging (MRI) has become the gold standard for the early detection of sacroiliitis, owing to its ability to visualize active inflammatory lesions, including bone marrow edema, synovitis, and capsulitis. Nevertheless, interpreting pediatric SIJ-MRIs requires caution, as physiological bone marrow changes and ongoing ossification in growing children can resemble inflammatory findings [2]. Moreover, despite its high sensitivity for inflammatory lesions, MRI has limitations in detecting structural changes such as erosions, sclerosis, and ankylosis, particularly in the early stages. The spatial resolution of MRI may be insufficient to detect small erosions or subtle cortical irregularities, which are often better visualized on computed tomography (CT) [3]. This limitation is especially relevant in pediatric cases, where structural lesions may evolve slowly. Advanced MRI sequences, such as 3D-MENSA, 3D-MERGE, and ZTE, address the limitations of conventional imaging. ZTE captures signals from tissues with ultra-short T2 relaxation times, such as cortical bone. This rapid acquisition generates CT-like osseous contrast. However, a known limitation of ZTE is its inability to reliably visualize active inflammatory markers, specifically bone marrow edema and fat metaplasia. To address this gap and evaluate a more comprehensive imaging protocol, 3D-MERGE and 3D-MENSA utilize multiple gradient echoes with 3D volumetric acquisitions. This technique provides high spatial resolution and superior contrast at bone cartilage and soft tissue interfaces. Clinically, these radiation-free sequences are increasingly used for high-fidelity evaluation of complex joint and spine pathologies. Most recently, a study showed that zero echo time (ZTE) imaging held promise for providing sacroiliac joint visualization comparable to low-dose CT scans for detecting erosions and sclerosis [4]. In this study, we aimed to assess advanced MRI sequences (including 3D-MENSA, 3D-MERGE, and ZTE) for detecting structural lesions of sacroiliitis, with pre-existing low-dose CT serving as the reference standard. As a secondary objective, we qualitatively evaluated the visibility of both active inflammatory lesions (bone marrow edema) and fat metaplasia to determine the feasibility of a comprehensive, “one-stop” radiation-free assessment.

## 2. Materials and Methods

### 2.1. Study Design and Population

This cross-sectional observational study included pediatric patients with juvenile idiopathic arthritis (JIA) as the ERA subtype. Patients were classified as having ERA according to the classification criteria established by the International League of Associations for Rheumatology (ILAR) [5]. Demographic characteristics (age, sex, family history), clinical features (inflammatory back pain, morning stiffness, enthesitis, uveitis), physical examinations (Schober’s test, Patrick-FABER test, sacroiliac joint tenderness), and laboratory parameters—including white blood cell (WBC) count, erythrocyte sedimentation rate (ESR), C-reactive protein (CRP), and HLA-B27 status—were systematically recorded. Disease activity was evaluated using the juvenile Spondyloarthritis Disease Activity Index (JSpADA), a validated instrument specifically developed to assess disease activity in patients with juvenile spondyloarthritis. Treatment modalities were also thoroughly reviewed. Patients diagnosed with psoriasis, familial Mediterranean fever, or inflammatory bowel disease were excluded to obtain a more homogeneous study group.

Sacroiliitis was initially suspected based on clinical findings suggestive of inflammatory back pain and/or limited spinal mobility according to ASAS criteria, and the diagnosis was confirmed by MRI in all patients prior to study inclusion. The study protocol was approved by the institutional ethics committee, and written informed consent was obtained from all participants and their parents.

### 2.2. Magnetic Resonance Imaging Procedure

All MRI scans were performed using a 1.5-T scanner (GE Healthcare, Milwaukee, WI, USA) with a 16-channel air coil. The total MRI examination time, comprising standard clinical and advanced sequences, was approximately 30 min. No sedation was required for any patient because of the manageable duration. The imaging protocol included the following routine clinical sequences: fat-suppressed axial oblique T2-weighted (TR/TE 2500/85 ms, echo train length 16), axial oblique T1-weighted (TR/TE 610/minimum ms, echo train length 3), coronal oblique T1W (TR/TE 590/minimum ms, echo train length 3), coronal oblique STIR (TR/TE 4250/42 ms, echo train length 16), and axial oblique T2-weighted (TR/TE 4650/85 ms, echo train length 16). Standard imaging parameters for routine sequences included a 2.5 mm slice thickness, 1 mm gap, 384  ×  384 matrix, and 26  ×  26 cm field of view. The ZTE sequence was also acquired (TR/TE 568/0 ms, 1.6 mm slice thickness, no gap, 280  ×  280 matrix, 25  ×  25 cm field of view, flip angle 2°, and scan time 2:55 min). The 3D-MENSA sequence (TR/TE 18/9 ms, 2 mm slice thickness, no intersection gap, a 256  ×  256 matrix, a 23 cm  ×  23 cm field of view, flip angle 12°, and scan time 3:25 min) and the 3D-MERGE sequence (TR/TE 40/17 ms, 3 mm slice thickness, no intersection gap, a 300  ×  300 matrix, a 23 cm  ×  23 cm field of view, flip angle 5°, and scan time 3:15 min) were also performed. The variation in slice thickness between the standard clinical T1W (2.5 mm) and advanced 3D sequences (1.6–3 mm) is an inherent protocol difference. Its potential impact on diagnostic performance is discussed as a study limitation.

### 2.3. Low-Dose Computed Tomography Protocol

No new CT scans were performed for the purpose of this study. To adhere strictly to radiation safety principles, we retrospectively utilized low-dose CT datasets acquired as part of a previous clinical evaluation protocol. The mean time interval between the reference low-dose CT and the new MRI scans was 2.5 ± 0.5 months. To rule out structural disease progression during this period, we compared baseline ZTE sequences (acquired alongside the low-dose CT) with the new MRI ZTE sequences. Patients with any structural interval changes (silent progression) were excluded from the analysis of diagnostic accuracy. CT imaging was performed using a 640-slice MSCT scanner (Canon Medical Systems, Otawara, Japan), with an average effective dose of 0.615 mSv per sacroiliac joint scan. Because conventional radiography is frequently inadequate in children, low-dose CT was used in accordance with the ALARA (As Low as Reasonably Achievable) principle, with an estimated radiation dose within the acceptable low-risk range (0.1–1 mSv) [6]. Scanning parameters included a tube voltage of 120 kV, a tube current automatically adjusted for patient size (80–95 mAs), a rotation time of 0.5 s, a slice thickness of 0.5 mm, a reconstruction interval of 2.5 mm, a window width of 2700 Hounsfield units (HU), and a window level of 350 HU. No intravenous contrast was administered.

### 2.4. Comparative Analysis of CT and MRI Scans

All MRI and CT scans were independently on anonymized images evaluated by two pediatric radiologists (T.O., 12 years of experience; Y.A., 27 years of experience), who were blinded to patient information. Each scan was independently evaluated by two readers, and interobserver agreement was assessed using a two-way mixed-effects model and the absolute-agreement form of the intraclass correlation coefficient (ICC), which was appropriate because continuous quantitative scores were analyzed.

The imaging evaluations were performed in a randomized sequence, with each reader assessing the scans in varying order across modalities to minimize potential bias. All images were anonymized and reviewed twice by each reader at separate time points. The order of patients was randomized for each assessment, and a 4-day interval was maintained between the first and second readings of each modality to minimize recall bias.

Structural lesions—including erosion, sclerosis, and joint-space changes—were scored in the coronal oblique plane. The sacroiliac joint was divided into anterior, middle, and posterior thirds [4,7] (Supplementary Figures S1–S3), and from each third, the section showing the most prominent structural change was selected, ensuring that selected sections were not adjacent. Although scoring was based on a representative section for each third, all sequences were comprehensively reviewed in multiple planes and slices for every patient to ensure accurate lesion identification. Specifically, the evaluating radiologists utilized the PACS workstation to dynamically generate multiplanar reconstructions (MPR) for all 3D sequences as needed, allowing them to confidently assess lesions in any desired orientation. While structural lesions were scored separately for each sequence (including conventional T1W), coronal oblique STIR, axial oblique fat-suppressed T2, and axial oblique T2 sequences served as complementary and supportive references during the evaluation. Fat metaplasia and bone marrow edema were evaluated qualitatively as secondary findings.

Structural lesions were defined as follows [4] (Supplementary Table S1):

Erosion: Cortical bone defects with adjacent marrow signal alteration. On ZTE images, erosions were defined as focal cortical interruptions, with fluid-sensitive sequences used as anatomical references when necessary.

Sclerosis: Subchondral sclerosis was defined as subchondral signal–density alterations greater than 5 mm in thickness, appearing hyperdense or high-signal on ldCT and ZTE MR images, and low-signal on 3D-MENSA, 3D-MERGE and T1-weighted sequences [8].

Joint-space changes: Narrowing, pseudo-widening (as an apparent widening of the sacroiliac joint space on MRI caused by adjacent erosions or irregularity of the subchondral bone, rather than true joint-space enlargement), or partial ankylosis visible on both MRI and CT.

Fat metaplasia: A well-demarcated area of increased signal intensity on T1W images within the subchondral bone marrow, showing signal suppression on fat-suppressed sequences.

Bone marrow edema: An area of increased signal intensity on fluid-sensitive fat-suppressed sequences (STIR or T2-weighted fat-suppressed images) within the subchondral bone marrow of the sacroiliac joint, with corresponding low signal intensity on T1W images.

### 2.5. Statistical Analysis

All statistical analyses were performed using IBM SPSS Statistics for Windows, version 29.0 (IBM Corp., Armonk, NY, USA). The Shapiro–Wilk test was used to assess the normality of continuous variables. As the normality assumption was not met, continuous data were reported as median (minimum–maximum), while categorical data were summarized as frequencies and percentages. Agreement between low-dose CT (reference standard) and different MRI sequences was assessed using the intraclass correlation coefficient (ICC) with 95% confidence intervals (CI). The intraclass correlation coefficient (ICC) was used in two contexts: to assess interobserver agreement between readers and to evaluate agreement between MRI sequences and low-dose CT as the reference standard. Agreement for erosion and sclerosis was evaluated at the quadrant level, and agreement for joint-space changes was at the joint level [7] (Supplementary Figures S1–S3). A *p* value of <0.05 was considered statistically significant. The sacroiliac joints were divided into 12 joint levels (6 for each side) and 24 quadrants (12 for each side). The total erosion and sclerosis scores were calculated by summing the scores from the 24 quadrants, and the total joint-space-alteration score was derived from the sum of the 12 joint levels.

## 3. Results

### 3.1. Demographic and Clinical Characteristics

A total of 23 patients diagnosed with JIA and classified as ERA with sacroiliitis were included in the study; 14 (60.9%) were male. The demographic and clinical characteristics of the patients are shown in Table 1.

At the time of radiologic evaluation, clinical sacroiliitis was present in 11 patients (47.8%), and 10 patients (43.5%) reported morning stiffness. At the time of clinical evaluation the median JSpADA was 2.75 (2–3.38). At this point, 14 (60.9%) patients were receiving disease-modifying anti-rheumatic drugs (DMARDs) (methotrexate = 12, sulfasalazine = 2) (median treatment duration: 16 months), and 17 (73.9%) were on biologic agents (etanercept = 13, adalimumab = 4) (median duration: 30 months). Follow-up imaging was performed for persistent symptoms in 11 patients and for assessment of treatment response—typically 6–12 months after a therapeutic change—in 12 patients.

### 3.2. Comparison of Images

The median total erosion scores were 5 (0–36) in low-dose CT, 4.5 (0–35) in ZTE, 4.5 (0–34) in 3D-MENSA, and 3.5 (0–31) in 3D-MERGE, while it was 0 (0–22) in the conventional T1W sequence (*p* < 0.001) (Table 2). The interobserver reliability for erosion scoring, as measured by the ICC, was excellent across the advanced radiologic techniques, with ICC values ranging from 0.924 to 0.998. ZTE-MRI demonstrated the highest reliability (ICC: 0.998), followed by 3D-MENSA (ICC: 0.996) and 3D-MERGE (ICC: 0.924). In contrast, the ICC for the conventional MRI T1W sequence was substantially lower at 0.71 (Table 3). All advanced radiologic techniques demonstrated strong agreements with low-dose CT in detecting sclerosis; the median total sclerosis score was 0 (range 0–12) (Table 3). The median score for joint-space changes was 2 (0–18) in low-dose CT, ZTE, and 3D-MENSA; 2 (0–17) in 3D-MERGE; and lower, 0 (0–14), in the MRI T1W sequence. Furthermore, interobserver agreement between two readers was excellent, with an ICC of 0.91 (95% confidence interval [CI]: 0.85–0.95).

### 3.3. Qualitative Assessments and Secondary Findings

**Figure 1:**
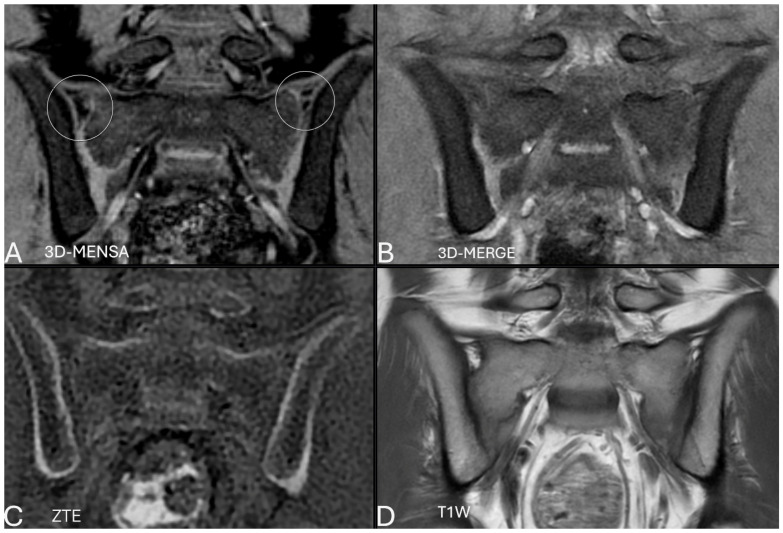
Coronal oblique MR images from a 12-year-old male patient were acquired from the same slice and time point using 3D-MENSA (**A**), 3D-MERGE (**B**), ZTE (**C**), and T1W (**D**) sequences. The 3D-MENSA (**A**) sequence allows for a clear distinction between the ligamentous component, the adjacent bone cortex, and the synovial component of the joint (white circles).

**Figure 2:**
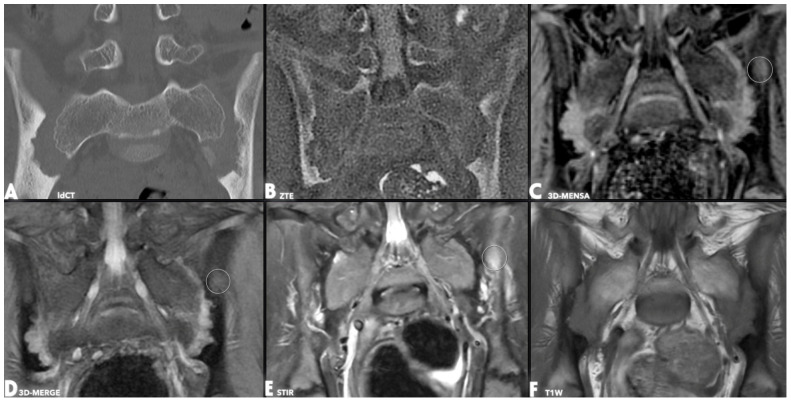
Coronal oblique images from the same slice and date in a 13-year-old patient comparing low-dose CT (**A**) and various MR sequences (**B**–**F**). Bilateral coalescent cortical erosions on the iliac sides and extensive adjacent subchondral sclerosis are observed. Subchondral sclerosis appears hyperdense on low-dose CT (**A**) and hyperintense on ZTE (**B**), whereas it is nearly signal-void on 3D-MERGE (**D**), 3D-MENSA (**C**), and T1-weighted (**F**) sequences. Erosions are sharply delineated on ZTE (**B**), 3D-MENSA (**C**), and 3D-MERGE (**D**) sequences, whereas they are visible but less detailed on the T1-weighted (**F**) image. A region of bone marrow edema in the left ilium is hyperintense on 3D-MENSA (**C**) with a signal comparable to STIR (**E**) and is also visible on 3D-MERGE (**D**) with lower hyperintensity (white circles).

**Figure 3:**
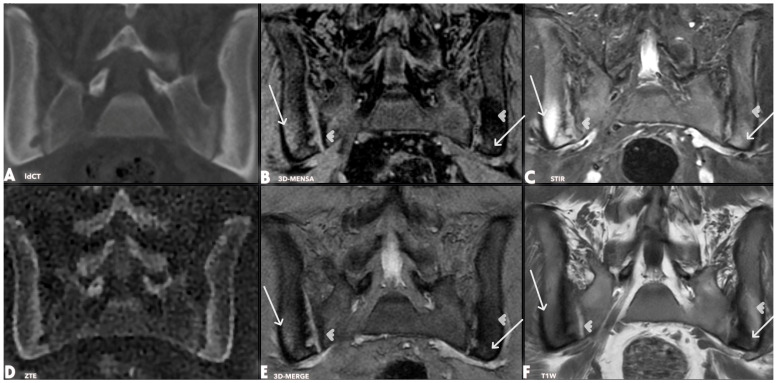
Coronal oblique images of a 17-year-old male patient were acquired from the same slice using low-dose CT (**A**), 3D-MENSA (**B**), STIR (**C**), ZTE (**D**), 3D-MERGE (**E**), and T1W (**F**) sequences. White arrowheads indicate fat metaplasia—hypointense on 3D-MENSA (**B**), hyperintense on T1W (**F**), and faintly visible on STIR (**C**) and 3D-MERGE (**E**); not detected on ZTE (**D**) or low-dose CT (**A**). White arrows show bone marrow edema, which appears hyperintense on 3D-MENSA (**B**) and STIR (**C**), slightly hyperintense on 3D-MERGE (**E**), and not visible on low-dose CT (**A**) or ZTE (**D**).

**Figure 4:**
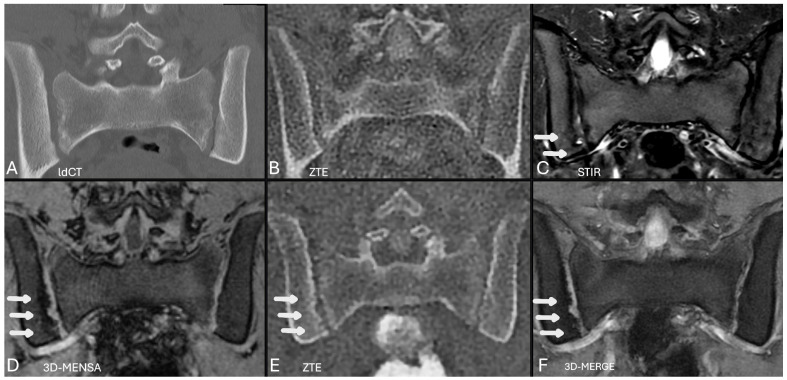
Coronal oblique ldCT, ZTE, STIR, 3D-MENSA, and 3D-MERGE images of a 14-year-old male patient at the same anatomical level. (**A**,**B**) Images obtained 3.5 months prior; show the absence of erosions on the initial ldCT and ZTE scans. (**C**–**F**) Current follow-up images. The current ZTE, 3D-MENSA, and 3D-MERGE images reveal newly developed cortical erosions on the iliac aspect (white arrows). Notably, no bone marrow edema is observed on the current STIR sequence.

A notable qualitative finding was that the 3D-MENSA sequence allowed for distinct differentiation of the ligamentous component of the sacroiliac joint from the synovial component and the adjacent bone cortex due to its high fluid-to-tissue contrast (Figure 1, Figure 2, Figure 3 and Figure 4).

Regarding secondary objectives, fat metaplasia was identified on T1W sequences in 7 of the 23 patients. In these cases, the same lesions were also visible on both 3D-MENSA and 3D-MERGE sequences. Subchondral bone marrow edema, indicating active sacroiliitis, was detected on STIR sequences in five patients. In all five cases, the edema was also visible on 3D-MENSA sequences, whereas 3D-MERGE sequences demonstrated the edema in four of the patients. Additionally, erosions and sclerosis were more prevalent on the iliac than on the sacral side.

## 4. Discussion

The principal finding of this study is that ZTE, 3D-MENSA, and 3D-MERGE demonstrate excellent interobserver reliability and high diagnostic accuracy for detecting erosions, sclerosis, and joint-space changes, with ZTE showing the highest performance. Additionally, 3D-MENSA effectively visualize fat metaplasia and bone marrow edema, suggesting its feasibility for a comprehensive, “one-stop” assessment of inflammatory and structural changes in sacroiliitis.

Sacroiliac involvement is a common feature of ERA, affecting up to 70% of individuals over time [9]. Despite its high frequency, the radiological evaluation has limitations due to the anatomical structure of the SIJ. Conventional T1W MRI sequences are limited in detecting cortical erosions, due to the low contrast between the joint space and cortical bone and to partial-volume (Figure 2 and Figure 3). Alternative imaging techniques increasingly address the limitations of traditional radiography, CT, and MRI in sacroiliac joint (SIJ) evaluation [10]. Although dual-energy CT (DECT) visualizes structural changes and edema, high radiation exposure restricts its pediatric use [11], and its sensitivity decreases near cortical bone or sclerotic areas [12]. VIBE sequences improve erosion detection at 1.2 mm slice thicknesses, though this advantage diminishes at 3 mm [13]; notably, our 2–3 mm gradient echo sequences outperformed T1-weighted imaging. Furthermore, while SWI effectively detects sclerosis and erosions [14], complex coil designs and interpretation challenges limit its routine musculoskeletal application [10]. Finally, synthetic CT offers clear anatomical differentiation but is constrained by strict tissue contrast and post-processing requirements [15].

Gradient echo (GRE) sequences offer increased contrast among the joint cavity, cartilage, and cortical bone, allowing improved visualization of cortical erosion. In our study, all GRE sequences performed at 2–3 mm demonstrated greater diagnostic utility than T1W sequences (Figure 2, Figure 3 and Figure 4).

While VIBE is the most widely studied 3D GRE sequence [16], 3D-MENSA [17,18] and 3D-MERGE [19,20] remain underexplored in SIJ imaging despite their use in other musculoskeletal regions. Rather than detailing their underlying physics, we highlight their clinical value, which lies in providing high spatial resolution, rapid image acquisition, and distinct tissue contrast: fluid-rich tissues appear hyperintense, whereas cortical bone appears hypointense. This generates high contrast between the joint cavity and the articular surfaces, facilitating the detection of bone marrow edema, subchondral sclerosis, and fat metaplasia. To our knowledge, this is the first study to evaluate the performance of these specific sequences in pediatric sacroiliitis using low-dose CT as the reference standard.

A critical advantage observed in our study was 3D-MENSA’s ability to differentiate between soft tissue components. Although other sequences can differentiate between joint components and the bone cortex, the 3D-MENSA sequence offers distinct clarity. The high signal intensity obtained from the synovial component creates a marked contrast difference between the adjacent bone cortex and the cartilaginous–synovial complex, enabling a much clearer visualization of the anatomical evaluation (Figure 1, Figure 2, Figure 3 and Figure 4). The ligamentous component of the SIJ is primarily situated in the superior aspect, coinciding with the S1 level. In the pediatric population, this region is characterized by ongoing ossification, often resulting in a normal cortical margin that is indistinct or absent [21]. Therefore, the high-contrast capability of the 3D-MENSA sequence is particularly important in this area, as it allows clear differentiation of ligamentous tissue from the immature bone cortex, thereby preventing the misinterpretation of physiological blurring as erosion.

We previously demonstrated that ZTE is highly sensitive in detecting cortical erosions, joint-space narrowing, and subchondral sclerosis [4]. However, both ZTE and low-dose CT fail to reliably detect fat metaplasia and bone marrow edema (Figure 2 and Figure 3). Fat metaplasia remains relatively understudied despite its potentially high prognostic value [21,22]. Although GRE sequences include fat suppression—raising concerns that this might limit the detection of fat metaplasia—the affected areas still appear distinctly darker than normal bone marrow, allowing for reliable identification [23]. Although differentiating fat metaplasia from subchondral sclerosis can be difficult, sclerosis typically appears almost completely dark (signal-void), which helps distinguish between the two. In clinical practice, T1W imaging is required to confirm the presence of fat metaplasia. 3D-MENSA complements this by providing high-resolution spatial mapping. Once T1W identifies the fatty tissue, 3D-MENSA precisely localizes these lesions relative to bone erosions and the true cortex, offering a comprehensive 3D evaluation that T1W alone lacks.

Previous studies evaluating older GRE sequences such as FLASH and DESS lacked robust reference standards or yielded suboptimal bone-joint contrast [24,25]. Furthermore, anatomical differences must be considered when interpreting SIJ imaging. In adults, the sacral side of the SIJ cartilage can reach a thickness of up to 4 mm, whereas the iliac-side measures 1–2 mm and exhibits greater cellular density [26]. Although this evidence is predominantly derived from adult populations, we believe this anatomical distinction remains a critical factor in the pediatric cohort. Indeed, our findings reflect this asymmetry, with a significantly higher prevalence of erosion and sclerosis on the iliac side compared with the sacral side (Figure 2, Figure 3 and Figure 4). Currently, there is no consensus on the optimal timing and frequency of MRI for monitoring sacroiliitis [27]. Notably, although the current literature regarding these imaging techniques is restricted to adult patients, our study specifically highlights their diagnostic utility in a pediatric population. Rapid, short, and cost-effective imaging protocols could allow for more frequent follow-up and early detection of treatment failure. A key strength of our study is the successful implementation of this advanced technique in pediatric patients, who are traditionally challenging to study because of compliance issues and motion artifacts.

A critical challenge in pediatric sacroiliitis is “silent progression,” where structural damage evolves despite clinical improvement or resolution of inflammation [28]. This underscores the need for safe, longitudinal monitoring. We observed an illustrative follow-up case (Figure 4) in which the comparison baseline and follow-up ZTE revealed the development of erosions over a 3.5-month period, despite resolution of bone marrow edema. These newly developed structural lesions were also clearly identified on the concurrent follow-up 3D-MENSA and 3D-MERGE sequences. This case was excluded from the statistical analysis because a concurrent follow-up CT was not ethically justifiable. Importantly, these subtle interval changes were clearly depicted by advanced sequences without the need for a follow-up CT scan, highlighting their profound clinical value for radiation-free monitoring of structural progression in pediatric patients, thereby directly addressing the threat of silent progression.

### Limitations

This study has several limitations. The sample size was small (*n* = 23) because of the specific nature of the study population. Furthermore, the majority of patients were receiving biologic treatment, resulting in limited number of samples with active inflammation. Second, inherent challenges in pediatric sacroiliac imaging, such as ongoing ossification and physiological cortical irregularities (especially at the S1 level), can complicate the assessment of structural lesions [21]. Third, the conventional T1-weighted sequences were acquired using a standard clinical protocol (2.5 mm slice thickness), whereas the advanced 3D sequences were acquired using thinner slices (1.6–2 mm). While this difference inherently disadvantages the T1W sequence and explains its lower performance, our aim was to demonstrate the diagnostic gain of adding these advanced sequences to the standard clinical routine. Regarding the reference standard, no new CT scans were performed; instead, we retrospectively utilized pre-existing low-dose CT datasets to adhere strictly to ethical radiation safety standards. Potential interval structural changes between CT and MRI scans were excluded by comparing baseline and current ZTE sequences [Figure 4].

## 5. Conclusions

In conclusion, 3D-MENSA and 3D-MERGE sequences effectively detect structural lesions, including erosions, sclerosis, and joint-space changes, with diagnostic accuracies comparable to low-dose CT. Furthermore, these sequences offer the promise of potentially visualizing active inflammation and fat metaplasia. Consequently, they could be integrated into routine MRI protocols to monitor pediatric sacroiliitis.

## Figures and Tables

**Table 1:** Demographic, clinical and laboratory findings, and treatments of patients.

Variables	
Current age, years, median (min–max)	14 (12–15)
Age at diagnosis, years, median (min–max)	10 (9–14)
Follow-up duration, months, median (min–max)	32 (19–86)
A family history of rheumatic disease, *n* (%)	17 (73.9)
History of parental consanguinity, *n* (%)	1 (4.3)
Back pain, *n* (%)	6 (26.1)
Morning stiffness, *n* (%)	19 (82.6)
Peripheral arthritis, *n* (%)	18 (78.3)
Heel pain, *n* (%)	11 (47.8)
Enthesitis, *n* (%)	9 (39.1)
Uveitis, *n* (%)	0 (0)
White blood cell count, mm^3^, median (min–max)	7406 (4500–12,590)
Hemoglobin, g/dL, median (IQR)	12.2 (8.7–14.2)
Platelet, mm^3^, median (IQR)	312,000 (201,000–573,000)
CRP, mg/dL, median (IQR)	2.5 (0.12–11)
ESR, mm/h, median (IQR)	20 (1–73)
NSAIDs, *n* (%)	23 (100)
DMARDs, *n* (%)	23 (100)
Biologic agents, *n* (%)	17 (73.9)

CRP, C-reactive protein; DMARDs, disease-modifying anti-rheumatic drugs; ESR, erythrocyte sedimentation rate; max, maximum; min, minimum; NSAIDs, non-steroidal anti-inflammatory drugs; IQR, interquartile range.

**Table 2:** Measurement of erosions, sclerosis, and joint-space changes across different radiologic techniques.

	Low-Dose CT	MRI-T1W	ZTE	3D-MENSA	3D-MERGE
**Total Erosion Scores**	5 (0–36)	0 (0–22)	4.5 (0–35)	4.5 (0–34)	3.5 (0–31)
**Iliac-Side Erosion Scores**	4 (0–29)	0 (0–20)	3.5 (0–29)	3.5 (0–30)	2.5 (0–28)
**Sacral-Side Erosion Scores**	1 (0–14)	0 (0–7)	1 (1–13)	1 (1–12)	0.5 (0–12)
**Total Sclerosis Score**	0 (0–12)	0 (0–12)	0 (0–12)	0 (0–12)	0 (0–12)
**Iliac-Side Sclerosis Score**	0 (0–12)	0 (0–12)	0 (0–12)	0 (0–12)	0 (0–12)
**Sacral-Side Sclerosis Score**	0 (0–0)	0 (0–0)	0 (0–0)	0 (0–0)	0 (0–0)
**Joint-space changes**	2 (0–18)	0 (0–14)	2 (0–18)	2 (0–18)	2 (0–17)

Parameters were given as median (minimum–maximum). Low-dose CT demonstrated the highest median total and iliac-side erosion scores, whereas MRI-T1W yielded consistently lower erosion scores. ZTE, MENSA, and MERGE sequences showed values comparable to CT. Sclerosis scores were uniformly zero across all modalities, and joint-space-change scores were similar between CT and advanced MRI sequences.

**Table 3:** Comparing the ability of various MRI sequences to low-dose CT in detecting structural lesions of the sacroiliac joints.

Structural Lesion	Comparison	ICC (95% CI)	*p* Value	Agreement
**Erosions**	Low-dose CT vs. MRI-T1W	0.71 (0.58–0.82)	<0.001	Good
	MRI-T1W vs. ZTE	1.00 (0.99–1.00)	<0.001	Excellent
	MRI-T1W vs. 3D-MENSA	1.00 (0.99–1.00)	<0.001	Excellent
	MRI-T1W vs. 3D-MERGE	0.92 (0.85–0.97)	<0.001	Excellent
	ZTE vs. 3D-MENSA	0.63 (0.45–0.77)	<0.001	Moderate
	ZTE vs. 3D-MERGE	0.64 (0.47–0.78)	<0.001	Moderate
	3D-MENSA vs. 3D-MERGE	0.76 (0.61–0.87)	<0.001	Good
**Sclerosis**	Low-dose CT vs. MRI-T1W	0.78 (0.65–0.87)	<0.001	Good
	MRI-T1W vs. ZTE	0.94 (0.89–0.97)	<0.001	Excellent
	MRI-T1W vs. MENSA	0.99 (0.97–1.00)	<0.001	Excellent
	MRI-T1W vs. MERGE	0.94 (0.88–0.97)	<0.001	Excellent
	ZTE vs. MENSA	0.90 (0.82–0.95)	<0.001	Excellent
	ZTE vs. MERGE	0.79 (0.67–0.88)	<0.001	Good
	MENSA vs. MERGE	0.88 (0.79–0.94)	<0.001	Excellent
**Joint-space changes**	Low-dose CT vs. MRI-T1	0.85 (0.74–0.92)	<0.001	Excellent
	MRI-T1W vs. ZTE	1.00 (0.99–1.00)	<0.001	Excellent
	MRI-T1W vs. MENSA	1.00 (0.99–1.00)	<0.001	Excellent
	MRI-T1W vs. MERGE	0.99 (0.97–1.00)	<0.001	Excellent
	ZTE vs. MENSA	0.86 (0.75–0.93)	<0.001	Excellent
	ZTE vs. MERGE	0.86 (0.75–0.93)	<0.001	Excellent
	MENSA vs. MERGE	0.88 (0.78–0.94)	<0.001	Excellent

CT, computed tomography; ICC, intraclass correlation coefficient; MRI, magnetic resonance imaging; ZTE, zero echo time. Intraclass correlation coefficients (ICC) were interpreted as follows: <0.50 poor, 0.50–0.75 moderate, 0.75–0.90 good, and >0.90 excellent agreement. Reference standard: low-dose CT. Overall, MRI sequences demonstrated good to excellent agreement for erosions, sclerosis, and joint-space changes, particularly between low-dose CT, MRI-T1W and advanced sequences (ZTE, MENSA, MERGE), whereas agreement between ZTE and MENSA and MERGE for erosions was comparatively moderate, with all comparisons reaching statistical significance (*p* < 0.001).

## Data Availability

The data presented in this study are available on request from the corresponding author. The data are not publicly available due to privacy and ethical restrictions regarding the pediatric study population.

## Supplementary Materials

The following supporting information can be downloaded at: https://www.mdpi.com/article/10.3390/diagnostics16060887/s1, Table S1: Scoring method for structural abnormalities of the sacroiliac joint. * These parameters were assessed at the quadrant level for erosions and sclerosis, and at the joint level for joint-space changes. * A total of 24 distinct joint surfaces were scored for sclerosis and erosion, and 12 joint levels were evaluated for joint space on three separate slices passing through the sacroiliac joint. Figure S1: Segmentation on coronal oblique images. * Three-dimensional localization of all 24 regions. The first eight quadrants capture changes in the anterior aspects of both sacroiliac joints, which are anterior to the slices depicting the sacral neuroforamina (<180° of the circumference of S2 is visualized). The true pelvis is seen in the center of the image. The second eight quadrants (numbered 9–16) subdivide the central portion of both sacroiliac joints, defined by the depiction of the anterior sacral foramina. The remaining quadrants (numbered 17–24) represent the posterior part of the joints, which is recognized by visualization of the entheseal joint compartment in the middle, and stretching to the posterior and inferior aspect of the joint. Proper oblique coronal slice orientation (parallel to the axis of the S2 vertebra) is crucial for this scoring system [7]. Figure S2: Segmentation on coronal oblique images. Figure S3: Segmentation on coronal oblique images.

## Abbreviations

The following abbreviations are used in this manuscript:

3D-MENSA	Three-Dimensional Multi-Echo in Steady-State Acquisition
3D-MERGE	Three-Dimensional Multiple-Echo Recombined Gradient Echo
ALARA	As Low As Reasonably Achievable
CRP	C-Reactive Protein
CT	Computed Tomography
DECT	Dual-Energy Computed Tomography
DESS	Double Echo Steady State
DMARDs	Disease-Modifying Anti-Rheumatic Drugs
ERA	Enthesitis-Related Arthritis
ESR	Erythrocyte Sedimentation Rate
FLASH	Fast Low Angle Shot
GRE	Gradient Echo
HLA	Human Leukocyte Antigen
HU	Hounsfield Units
ICC	Intraclass Correlation Coefficient
CI	confidence intervals
ILAR	International League of Associations for Rheumatology
JIA	Juvenile Idiopathic Arthritis
MEDIC	Multiple-Echo Data Image Combination
MFFE	Multiple-Echo Data Image Combination (Philips Healthcare terminology)
MPR	Multiplanar Reconstruction
MRI	Magnetic Resonance Imaging
MSCT	Multi-Slice Computed Tomography
SIJ	Sacroiliac Joint
SNR	Signal-to-Noise Ratio
STIR	Short Tau Inversion Recovery
SWI	Susceptibility-Weighted Imaging
TE	Echo Time
TR	Repetition Time
UTE	Ultrashort Echo Time
VIBE	Volumetric Interpolated Breath-hold Examination
WBC	White Blood Cell
ZTE	Zero Echo Time
