# Supplementary material for: Advanced MRI Sequences for Structural Lesion Assessment in Sacroiliitis
Source: Diagnostics (Basel). 2026 Mar 17;16(6):887. doi: 10.3390/diagnostics16060887 (PMC13024943; doi:10.3390/diagnostics16060887)

Supplementary Table S1. Scoring method for structural abnormalities of the sacroiliac joint.

	Erosion	Sclerosis	Joint space changes
0	No erosion	No sclerosis or limited sclerosis (less than 5 mm)	No joint space changes
1	Small isolated erosions (1–2)	Evident sclerosis (≥5 mm)	Questionable widening or Narrowing
2	Definite erosions (3–5; <3 mm) or larger single erosion (>3 mm)		Pseudowidening
3	Multiple (>5) or confluent erosions		Partial ankylosis
4			Extensive/total ankylosis

*These parameters were assessed at the quadrant level for erosions and sclerosis, and at the joint level for joint space changes

*A total of 24 distinct joint surfaces were scored for sclerosis and erosion, and 12 joint levels were evaluated for joint space on three separate slices passing through the sacroiliac joint.

Supplementary Figure S1. Segmentation on coronal oblique images.

* Three-dimensional localisation of all 24 regions. The first 8 quadrants capture changes in the anterior aspects of both sacroiliac joints, which are anterior to the slices depicting the sacral neuroforamina (< 180° of the circumference of S2 is visualised). The true pelvis is seen in the centre of the image. The second 8 quadrants (numbered 9–16) subdivide the central portion of both sacroiliac joints, defined by the depiction of the anterior sacral foramina. The remaining quadrants (numbered 17–24) represent the posterior part of the joints, which is recognised by visualisation of the entheseal joint compartment in the middle, and stretching

to the posterior and inferior aspect of the joint. Proper oblique coronal slice orientation (parallel to the axis of the S2 vertebra) is crucial for this scoring system[7]

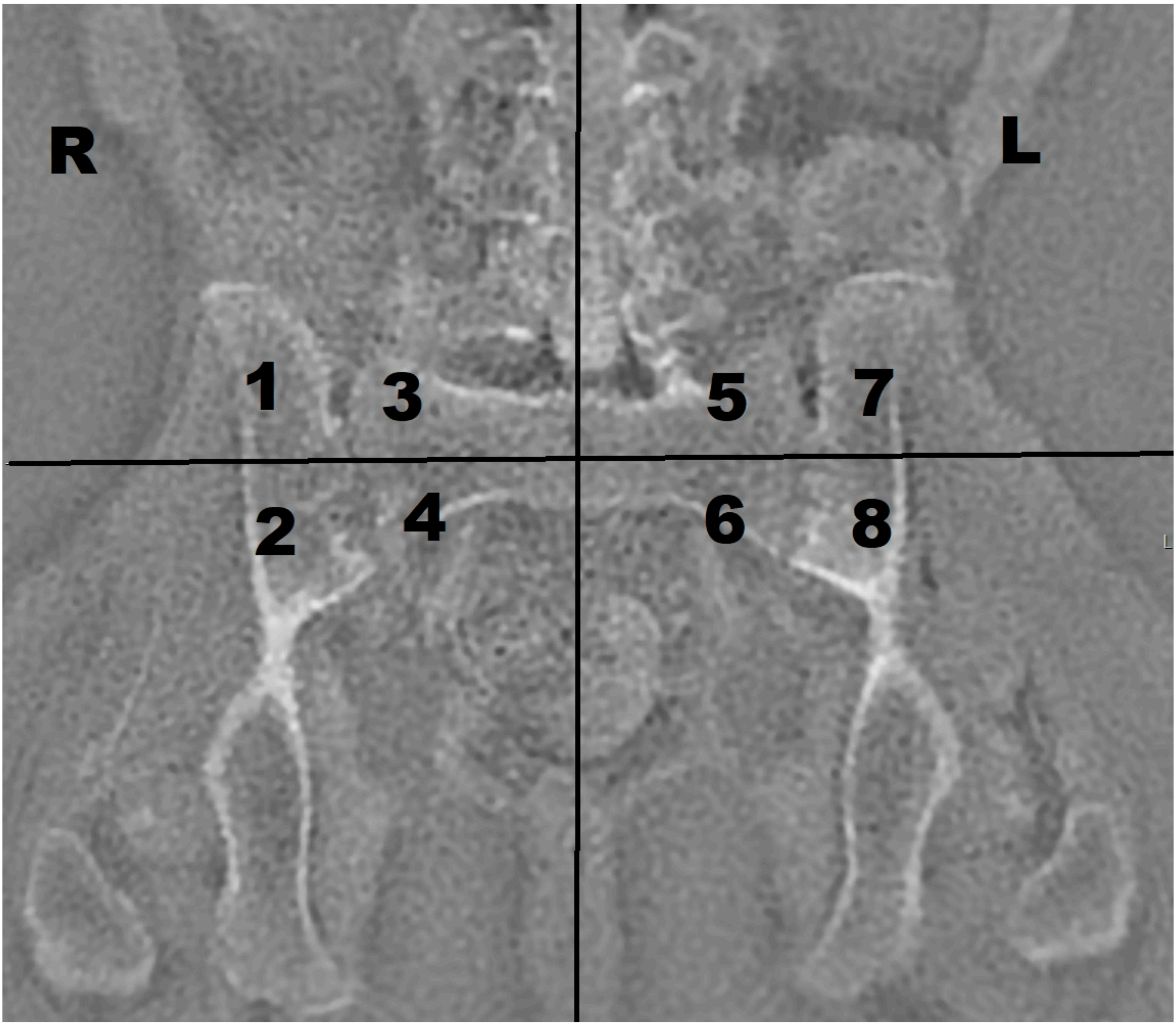

Supplementary Figure S2. Segmentation on coronal oblique images.

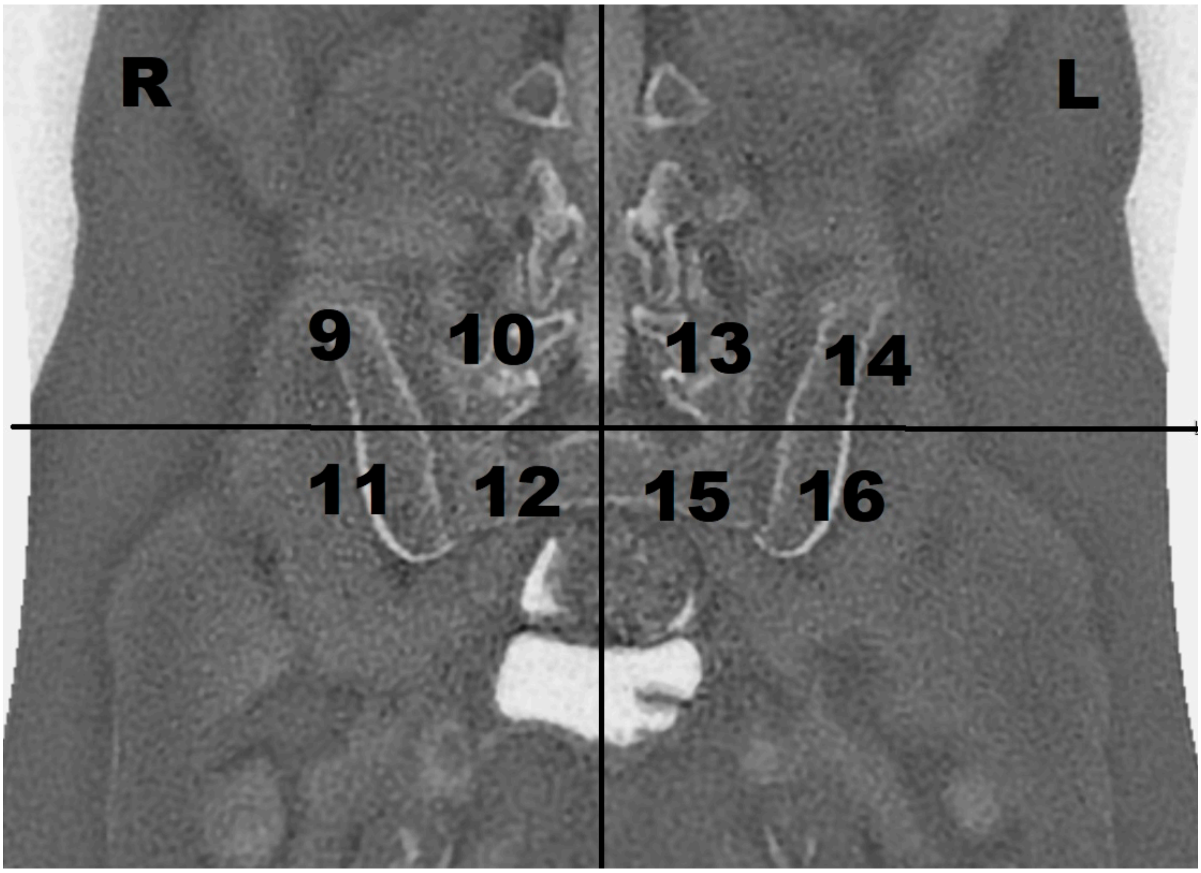

Supplementary Figure S3. Segmentation on coronal oblique images.

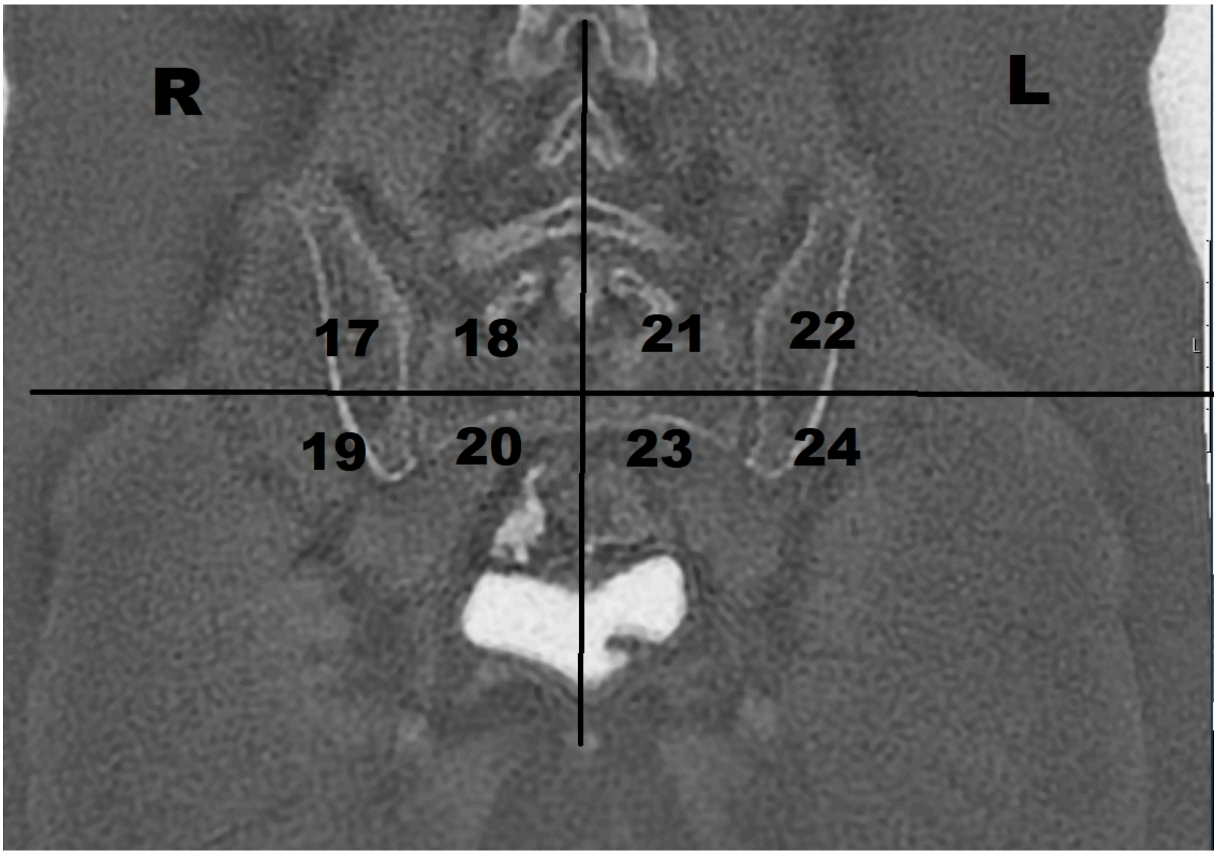

Supplement: Supplementary file 1 [file diagnostics-16-00887-s001.zip › diagnostics-4136222-supplementary.pdf]
